# Regulation of cell cycles is of key importance in human papillomavirus (HPV)- associated cervical carcinogenesis

**DOI:** 10.1590/S1516-31802003000300009

**Published:** 2003-05-01

**Authors:** Sylvia Michelina Fernandes Brenna, Kari Juhani Syrjänen

**Keywords:** Cervical cancers, Cell cycle, Human papillomavirus, Tumor suppressor genes, Histone deacetylase, Câncer cervical, Ciclo celular, Papilomavírus humano, Genes supressores de tumor, Histona deacetilase

## Abstract

The rapid progress in molecular biology has allowed the identification of the genes involved in different functions of normal cells and has also improved our understanding of the mechanisms of human carcinogenesis. The human papillomavirus (HPV) is a small double-stranded DNA tumor virus and its genes can manipulate cell cycle control to promote viral persistence and replication. The E6 and E7 proteins of high-risk HPV bind to cell cycle regulatory proteins and interfere with both G1/S and G2/M cell cycle checkpoints much more effectively than the low-risk HPV. The difference between the ability of low and high-risk HPV types to induce immortalization and transformation may well lie in their abilities to interact with the various cell cycle components, resulting in the loss of multiple cell cycle checkpoints, which are important in host genome fidelity, thus potentially resulting in accumulation of genetic abnormalities. Cervical cancer is one of the leading malignancies in women worldwide, with substantial morbidity and mortality. According to current concepts, HPV is recognized as the single most important causal agent in the pathogenesis of this cancer. HPV infection clearly precedes the development of malignancy, while being regularly associated with cervical cancer precursor lesions (all grades of squamous intraepithelial lesions). HPV-infected low-grade squamous intraepithelial lesion (SIL) has three possible outcomes: a) it may regress; b) it can persist; or c) it can make a clinical progression to in situ or invasive carcinoma. It has been well established by prospective cohort studies that the spontaneous regression rate increases in parallel with follow-up duration. In contrast, the clinical progression of lesions usually takes place quite rapidly, i.e. during the first two years from diagnosis. The mechanisms responsible for this divergent clinical behavior of HPV-associated squamous intraepithelial lesions are largely unknown, but currently under intense study in different laboratories worldwide.

## RESUMO

O rápido progresso dos estudos em biologia molecular permitiu identificar os genes envolvidos em diferentes funções celulares e também melhorou nossa compreensão sobre os mecanismos da carcinogênese humana. O papilomavírus humano (*human papillomavirus*, HPV) é um vírus de DNA e os seus genes podem manipular o controle do ciclo celular para promover a sua persistência e replicação. As proteínas E6 e E7 dos HPVs de alto risco oncogênico ligam-se às proteínas reguladoras do ciclo celular e interferem nas fases G1/S e G2/M mais efetivamente do que os HPVs de baixo risco. Os HPVs de baixo e alto risco diferem em sua capacidade de induzir imortalização e transformação celular bem como de interagir com os vários componentes de ciclo celular, o que resulta na perda de pontos de checagem do DNA, importantes para a manutenção do genoma do hospedeiro, e também resulta no acúmulo de anormalidades genéticas. O câncer de colo de útero é um dos principais cânceres genitais em mulheres em todo o mundo, com significativa morbidade e mortalidade. De acordo com conceitos atuais, o HPV é reconhecido como o agente causal mais importante na patogênese deste câncer. A infecção por HPV está associada a todas as lesões intra-epiteliais escamosas do colo do útero. A lesão intra-epitelial escamosa (*squamous intraepithelial lesion,* SIL) de baixograu tem três possíveis resultados: a) pode regredir; b) pode persistir ou c) pode progredir para câncer *in situ* ou invasivo. Estudos de coorte mostraram que a taxa de regressão espontânea destas lesões aumenta conforme o tempo de seguimento, em contraste com as lesões destinadas a progressão, que normal-mente evoluem rapidamente, geralmente nos primeiros dois anos. Os mecanismos responsáveis pelo comportamento clínico da lesão intra-epitelial escamosa associada ao HPV ainda não são totalmente conhecidos, mas atualmente têm sido motivo de estudos em todo o mundo.

## THE CELL CYCLE AND ITS REGULATION

Since the discovery of the deoxyribonucleic acid (DNA) structure, there has been a revolutionary improvement in our knowledge of normal cell functions. The DNA structure is a double-stranded helical molecule composed of two nucleotide chains connected by four nitrogenous bases: adenine (A), thymine (T), guanine (G) and cytosine (C). The DNA code is transmitted when DNA strands are copied during the cell cycle.^1^ Thus, the replication and division of a cell into genetically identical daughter cells depends on four steps, namely the G1 (gap), S (synthesis), G2 and M (mitosis) phases of the cell cycle. During the G1 phase, the cell accumulates cytoplasmic materials to duplicate the DNA. At the first stop of the cell cycle (named the R checkpoint), checking of the DNA status takes place, before cycle progression. In the event of any abnormality in the genetic information, this must be repaired first, and in such cases cell cycle arrest takes place. In the next steps, named the S and G2 phases, DNA replicates and the materials needed for cell duplication are obtained, respectively. The last step in the cell cycle is called the M phase, in which the cell duplication takes place.^1^

Cell cycle progression is controlled by a large group of regulatory proteins named cyclin-dependent kinases (CDKs). The active forms of these enzymes only appear in the form of complexes with specific proteins (active in a specific phase of the cycle) known as cyclins. There is often interaction with other proteins such as proliferating cell nuclear antigen (PCNA) and CDK inhibitors. The transitions in the cell cycle take place when the enzymatic activity of a given kinase activates the proteins required for progression from one stage of the cycle to the next. After the division of the cell, the DNA code is transcribed in the nucleus, to messenger ribonucleic acid (mRNA). The latter transfers the genetic information into the cytoplasm, where transfer RNA (tRNA) and synthesis RNA (sRNA) will be responsible for the synthesis of the proteins in the ribosomes. Each cell is programmed for specific functions and finishes its life cycle through apoptosis, the genetic control for removing inappropriate or senescent cells.^2^

This new understanding of the regulation of normal cell functions has significantly contributed to our concepts of molecular mechanisms in human carcinogenesis. In this review, we give a brief account of the role of human papillomavirus (HPV) as the single most important etiological agent of cervical cancer, by describing the molecular mechanisms whereby this tumor virus interferes with the regulation of the normal cell cycle.

## TUMOR SUPPRESSOR GENES

Tumor suppressor genes encode for proteins that regulate cell growth, and prevent the events that lead to malignant transformation of the cells. The first tumor suppressor gene ever cloned was named the Rb gene because it was first identified in retinoblastoma. The Rb gene is located on chromosome 13 and encodes a nuclear protein that regulates gene expression. Loss of the pRb pathway function certainly leads to loss of normal inhibitory controls of the cell cycle progression.^1^

**Figure 1:**
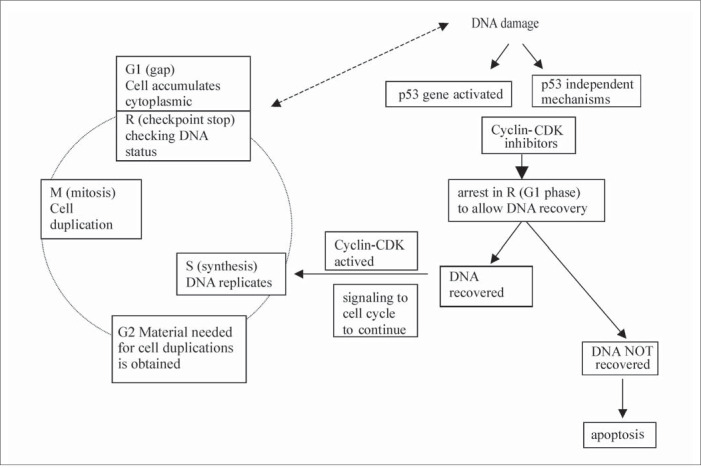
Synopsis of cell cycle regulation. CDK = Cyclin - dependent Kinases.

Another key tumor suppressor gene is the p 53 gene, also known as "the guardian of the genome ", which is located on the short arm of chromosome 17. This happens to be the most frequently mutated gene in human cancers. The p 53 gene was so named because it encodes a 53-kilodalton (kd) nuclear phosphoprotein that is normally present in very low quantities and has a very short half-life in normal cells. When DNA is damaged, however, the p53 gene is activated and the p53 protein interacts with other proteins called CDK/cyclin inhibitors, including the p16, p27 and p21^waf1cip1^. This concerted action results in the arrest of the cell cycle at the point R, in the G1 phase, to allow the DNA to recover. If the DNA repair is successful, the p21 signals to the CDK/cyclin compound for the cell cycle to continue (Figure 1). In cases where DNA repair is not possible, the p 53 protein signals to other regulatory proteins, such as bax, bcl-2 and c-myc, resulting in the induction of apoptosis, which eliminates cells with inappropriate genetic information.^1,3^ Thus, the p53 is considered to be a checkpoint control factor (Figure 1).

The mutations of the p53 gene have been extensively studied and described in several human malignancies, including cervical cancer.^4^ In such cases, the p53 gene can lose its functions, e.g. by deletion of one of its alleles (loss of heterozygosity). The cell cycle cannot arrest in the G1/S phase and continued replication of the DNA-damaged cells is allowed, thus leading to genome instability and accumulation of mutations.^3,5^ The detection of p53 protein using immunohistochemistry has been studied as a prognostic factor in invasive cervical squamous cell carcinoma.^6^

Polymorphisms of the p53 gene seem to be common and have been described in cervical cancer patients as well. People can carry one of two variations of the p53 gene in codon 72; p53 arg or p53 pro. It has been suggested that HPV oncoprotein (E6) more easily inactivates p53 arg (72) than pro (72), thus bearing some association with the outcome of HPV infections. Indeed, it has been proposed (although not unanimously agreed yet) that people who are homozygous to p53 arg might be less protected against the effects of oncogenic HPV types.^7^

## HUMAN PAPILLOMAVIRUS (HPV)

**Figure 2:**
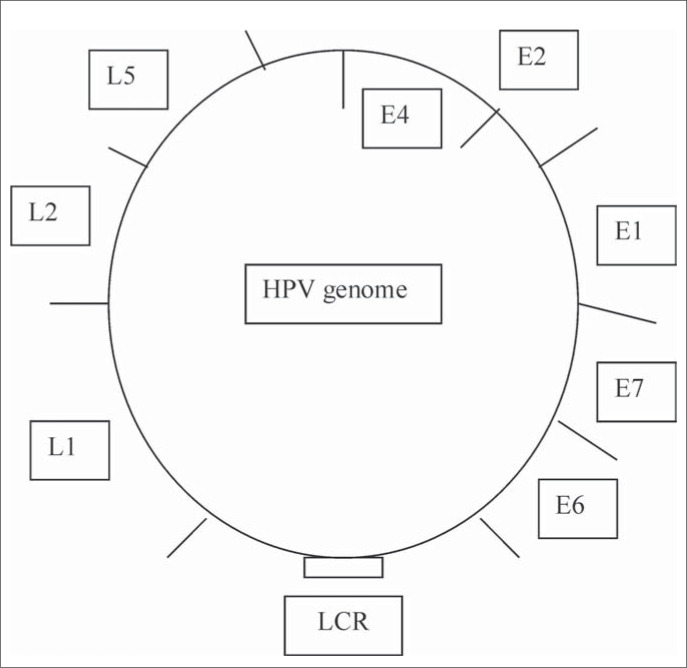
Organization of the human papillomavirus (HPV) genome.

HPVs are small DNA tumor viruses of approximately 55 nm in diameter, and over 100 different HPV types and many more sequences that are less well characterized have been isolated. HPVs are members of the Papovaviridae family. The mature HPV particle has an icosahedral capsid composed of two structural proteins: the L1 protein comprises 80% of the total viral protein; the L2 protein is a minor component. Contained within the capsid is the viral genome, which is a circular double DNA strand of approximately 7.9 kb in length, of which only one strand encodes the open reading frames (ORFs). ORFs are classified as early (E) or late (L), depending on the time point when the gene function occurs in the life cycle of the HPV infection (Figure 2). Early genes are expressed at the onset of the infection and mediate specific gene functions, controlling viral DNA transcription and replication and, in the case of oncogenic viruses, cell transformation as well.^3,8^

The E1 and E2 genes are involved in viral replication and genome maintenance. E1 has helicase activity that catalyzes the unwinding of the DNA duplex. It also brings the DNA polymerase to the origin of replication (*ori*), where the E1 and E2 proteins will initiate the replication. E2 also acts as a transcription repressor of the HPV E6 promoter. Although the E4 protein is a product of early gene expression, produced as a fusion protein incorporating part of the E1 protein (E1E4), it is often considered to be a late protein with production and localization in the cytoplasm of the upper epithelial layers just prior to full viral assembly.^9^ The E5 gene product interacts with cell membrane growth factors and is thought to play a role in transformation. However, the E6 and E7 genes encode the main transforming proteins. These are capable of immortalization and neoplastic transformation under appropriate conditions. The late genes L1 and L2 encode the structural proteins of viral particles that are expressed at the final stages of viral production. The finding that the E6 protein from high-risk HPV can induce the degradation of p53 either *in vitro* or *in vivo* has led to the proposal that such an inactivation pathway could be involved in the neoplastic process leading to cervical cancer.^3,10^

HPVs are epitheliotropic by nature and their life cycle is closely linked to the terminal differentiation of the squamous cells. In the cervix, initial infection is thought to occur in the epithelial basal cells, through small abrasions in the tissue or during squamous meta-plasia in the transformation zone when the basal cells are exposed.^11^ Once HPV has entered the target cells, it can remain latent or adopt replication in the nucleus, terminating in the synthesis and liberation of infective viral particles from the superficial cells.

The physical state of viral DNA in benign and malignant (and precancer) lesions is different. In the former, HPV DNA remains circular and does not integrate in the cell genome (i.e. it remains episomal). The other form of infection (non-permissive transforming infection) occurs when viral replication and vegetative viral production do not occur. This can take place in both squamous and glandular epithelia. However, infection of the cells that are committed to glandular differentiation and do not allow permissive HPV infection results in either aborted or non-permissive transformable infection. Viral DNA persists as either an extra-chromosomal element or integrates into the host DNA as a single copy or multiple head-to-tail tandem repeats, often at chromosomally fragile sites.^9^

## HPV AND THE CELL CYCLE

### HPV transcript in low-grade lesions

In HPV 6-induced condylomas, E6 is intensely expressed in the basal layers, whereas in the upper differentiated layers of condylomatous epithelium, no expression for E6 and E 7 is detected. The bulk of the cytoplasmic signals in the middle and upper third of the epithelium appears to represent E1-E4 mRNA. This is usually more abundant than transcripts from the late genes L1 and L2, which are only present in terminally differentiated cells in the superficial layers of the epithelium. The E1 and E2 signals are mostly detected in the nuclei, indicating that the levels of translatable mRNA with a coding potential for these early proteins are very low in benign lesions. E4 seems to be co-located with L 1, which is in agreement with the known functions of the E4 protein, thereby leading to the collapse of the cytokeratin network.^3,9^

### HPV transcripts in high-grade lesions and cancer

In high-grade lesions, the transcription of E 6/E7 is derepressed and the signals are detected throughout the whole undifferentiated epithelium. The transcription pattern is similar for both premalignant and malignant lesions. The overall low level of E6 mRNA expression and increase in E6/E7 transcripts indicates that mRNA with coding potential for E7 is expressed at higher levels. mRNA encoding the full-length E2 protein is usually missing in HPV 16-positive carcinoma with an intact E region, indicating the down-regulation of this transcription repressor. As the grade of the lesion increases, the L2 and L1 transcripts seem to vanish, although the L1-specific signals can still be detected in invasive carcinomas as well. The presence of L1 might reflect high differentiation of the carcinoma.^9^

### Deregulation of the cell cycle by E6 and E7

It is now well established that a number of HPV genes can manipulate cell cycle control to promote viral persistence and replication. The E6 and E7 proteins of the high-risk HPVs bind to cell cycle regulatory proteins and interfere with both the G1/S and G2/M cell cycle checkpoints, more effectively than the E6 and E7 proteins of the low-risk HPVs. In-vivo, numerous chromosome abnormalities have been identified in low-grade cervical lesions infected with high-risk HPVs, but not in those infected with the low-risk viruses. This correlates with the in-vitro observations that both HPV 16 E6 and E7 can alter cell cycle control and induce chromosome abnormalities in normal epithelial keratinocytes and fibroblasts. In addition, the high-risk HPV proteins can: 1) up-regulate expression of cyclins A and B in association with immortalization; 2) up-regulate cyclin E expression, shown recently to induce genetic instability; and 3) abrogate cyclin D1 expression, important in the Rb pathway.^10^

The differences between the ability of the low and high-risk HPV types to induce immortalization and transformation therefore may well lie in their abilities to interact with the cell cycle components, resulting in the loss of multiple cell cycle checkpoints that are important in maintaining host genome fidelity and thus leading to potential accumulation of genetic abnormalities.^3,9,10^

## HISTONE DEACETYLASE (HDAC)

Histone deacetylases (HDAC) are active components of the transcription co-repressor complexes. Currently, six HDAC enzymes are known in the human cell.^12^ Chromatin remodeling through HDAC activity is emerging as an important mechanism by which the gene transcription is regulated. Actively transcribed genes show a high level of histone acetylation, while repressed genes do not. It has been demonstrated that Rb can associate with HDAC, and both co-operate in repressing the transcription from E2F-regulated genes. These observations suggest that HDAC complexes are potential targets of viral oncoproteins .^12^

In addition, there could be a synergistic enhancement of the transactivation function by at least two different pathways; a) core his-tone acetylation and b) p53 acetylation. Hyperacetylation of histones correlates with enhanced transcription, presumably by increasing the accessibility of the transcription factors to nucleosomal DNA. Thus, the role of HDAC in the down-regulating of p53 seems to be HDAC dosage-dependent.^13^

## CERVICAL CANCER

Cervical cancer is the second most frequent malignancy in women (the first is breast cancer), and is responsible for substantial morbidity and mortality worldwide. Age-standardized incidence rates (ASIR) range from about 10 per 100,000 in most developed countries to more than 40 (and up to 100) per 100,000 in many developing countries.^14^

It is generally agreed that HPV is the single most important etiological agent involved in the pathogenesis of cervical cancer. HPV infection clearly precedes the development of malignancy, while being regularly associated with cervical cancer precursor lesions (all grades of squamous intraepithelial lesions). Usually, low-risk HPVs cause benign warts and have no oncogenic potential. On the other hand, high-risk HPVs are the causative agents of cervical cancer and its precursor lesions. The HPV types particularly associated with this disease include: HPV 16, 18, 31, 33, 35, 39, 45, 51, 56, 58, 59 and 68. There also appear to be variations in this risk, related to lower social class, cigarette smoking and the characteristics of male partners (a history of early sexual intercourse and many partners).^2,8,15^

It is well established by prospective cohort studies that cervical precancer lesions (cervical intraepithelial neoplasia, CIN) may regress, persist or progress to *in situ* or invasive carcinoma. However, the spontaneous regression rate increases in parallel with follow-up duration .^2,8^ Moreover, lesions destined for clinical progression do so quite rapidly and practically always during the first two years of follow-up, in contrast to lesions undergoing spontaneous regression, which can be a slow process .^2^ Spontaneous regression is frequent among women aged less than 35 years. In such cases, the HPV infection is transient, most probably because the woman's cell-mediated immune system is capable of eradicating the infection. In contrast, HPV infections are less frequent among women aged 35 years or more, and in these women, the infections are more often persistent and have higher potential for progression to high-grade CIN.^15^

These factors have important implications for the interpretation of follow-up data from different cohort studies that were run for relatively short lengths of time. Data sets from different cohort studies with up to 18 years of follow-up have described spontaneous regression rates of 56.7%, 50.4% and 12.2 % from HPV-CIN 1, 2 and 3, respectively. The progression to *in situ* carcinoma was 14.2%, 22.4% and 64% to HPV-CIN 1, 2 and 3, respectively (Table 1).^2^ These natural history data clearly suggest that the clinical behavior of CIN 2 is far closer to that of CIN 1, thus justifying the classification of both lesions in the low-grade category. This would differ from the current Bethesda System, which groups CIN 2 with CIN 3 as high-grade squamous intraepithelial lesions.

**Table 1:** Natural history of human papillomavirus-cervical intraepithelial neoplaisa (HPV-CIN) lesions. Adapted from Syrjänen.^2^ Based on cohort studies with up to 18 years of follow-up

	Regression (%)	Progression (%)
HPV-CIN 1	56.7	14.2
HPV-CIN 2	50.4	22.4
HPV-CIN 3	12.2	64

It would seem to be unnecessary to state, in conclusion, that the mechanisms responsible for this divergent biological behavior of HPV-associated squamous intraepithelial lesions are largely unknown. Nonetheless, such mechanisms are currently under intense study in different laboratories worldwide.

## References

[1] Kastan MB, De Vita VT, Helman S, Rosenberg S (1997). Molecular biology of cancer: the cell cycle. Cancer: principles & practice of oncology.

[2] Syrjänen K, Singer A, Monaghan JM (2000). Human papillomaviruses in pathogenesis of lower genital tract neoplasia. Lower genital tract precancer.

[3] Syrjänen SM, Syrjänen KJ (1999). New concepts on the role of human papillomavirus in cell cycle regulation. Ann Med.

[4] dos Santos Oliveira L do H, Fernandez A de P, Xavier BL, Machado-Rodrigues E de V, Cavalcanti SM (2002). Analysis of the p53 gene and papillomavirus detection in smears from cervical lesions. Sao Paulo Med J.

[5] (2001). IARC database of p53 gene mutations in human tumors and cell lines: updated compilation, revised formats and new visualization tools.

[6] Brenna SM, Zeferino LC, Pinto GA (2002). P53 expression as a predictor of recurrence in cervical squamous cell carcinoma. Int J Gynecol Cancer.

[7] Makni H, Franco EL, Kaiano J (2000). P53 polymorphism in codon 72 and risk of human papillomavirus-induced cervical cancer: effect of inter-laboratory variation. Int J Cancer.

[8] Syrjänen KJ, Fenoglio-Preiser CM, Wolff M, Rilke F (1992). Genital human papillomavirus infections and their associations with squamous cell cancer: reappraisal of the morphologic, epidemiologic and DNA data. Progress in surgical pathology.

[9] Syrjänen KJ, Syrjänen SM, Syrjänen KJ, Syrjänen SM (2000). Molecular biology of papilloma-viruses. Papillomavirus infections in human pathology.

[10] Southern SA, Herrington CS (2000). Disruptions of cell cycle control by human papillomaviruses with special reference to cervical carcinoma. Int J Gynecol Cancer.

[11] Murta EF, Souza MA, Araújo E, Adad SJ (2000). Incidence of Gardnerella vaginalis, Candida sp. and human papillomavirus in cytological smears. São Paulo Med J.

[12] Nguyen DX, Westbrook TF, McCance DJ (2002). Human papillomavirus type 16 E7 maintains elevated levels of the cdc25A tyrosine phosphatase during deregulation of cell cycle arrest. J Virol.

[13] Juan LJ, Shia WJ, Chen MH (2000). Histone deacetylases specifically down-regulate p53-dependent gene activation. J Biol Chem.

[14] Ferenczy A, Franco E (2002). Persistent human papillomavirus infection and cervical neoplasia. Lancet Oncol.

[15] Ferenczy A, Franco E (2001). Cervical-cancer screening beyond the year 2000. Lancet Oncol.

